# Correlation of anthropometric variables, conditional and exercise habits in activite olders

**Published:** 2012-09-30

**Authors:** Hilario Moreno Bolívar, Santiago Ramos Bermúdez, José H Parra Sánchez

**Affiliations:** a Universidad de Caldas, Manizales Colombia. E-mail: hilario.moreno@ucaldas.edu.co, santiago.ramos@ucaldas.edu.co; b Universidad Nacional de Colombia. E-mail:jhparrasa@unal.edu.co

**Keywords:** Exercise, elderly, correlation, anthropometry, functionality

## Abstract

**Objective:**

This study sought to correlate the anthropometric and functional variables, and exercise habits in a group of elderly adults who regularly attend exercise programs.

**Method:**

Participation of 217 subjects between 60 and 85 years of age, from 13 regions of Colombia. Anthropometric and functional assessment was conducted as a questionnaire on exercise habits.

**Results:**

Negative correlations were shown between exercise habits and body fat and positive correlations between hand strength and VO2 max. (r = 0.4), age was negatively associated to functional variables.

**Conclusions:**

The functional capacity is influenced by increased age and body fat. With higher frequencies of physical exercise, VO2 max. and strength improved, but less body fat was observed.

## Abstract (es)

**Objetivo:**

Este estudio tuvo como finalidad correlacionar las variables antropométricas, funcionales y hábitos de ejercicio físico en un grupo de adultos mayores que asisten regularmente a programas de ejercicio físico.

**Método:**

Participación de 217 sujetos entre 60 y 85 años de edad, procedentes de 13 regiones de Colombia. Se realizó una valoración antropométrica y funcional, así como un cuestionario sobre hábitos de ejercicio.

**Resultados:**

Se presentaron correlaciones negativas entre hábitos de ejercicio y grasa corporal, como positiva entre fuerza de la mano y Vo2 máx. (r = 0.4), la edad se asocia negativamente con las variables funcionales.

**Conclusiones:**

La capacidad funcional se ve influenciada por el incremento de la edad y grasa corporal. A mayor frecuencia de ejercicio físico se observa un mejor VO2 máx. y fuerza pero menor grasa corporal.

## Introduction

Aging implies structural and functional changes in humans; these appear in physical and mental aspects, generating deterioration that is reflected in lower capacity to perform basic activities of daily life (BADL) like eating and showering; in advanced activities of daily life (AADL) like walking and climbing stairs; inabilities that generate functional limitation, being a factor that causes detriment in the quality of life in the enderly^1^. 

Added to these inevitable modifications is a phenomenon regularly present in the elderly like overweight conditions, which if not controlled, worsens problems of functionality and independence that impact upon their morbidity and mortality. But it is also true that physical exercise programs developed with scientific rigor, bearing in mind anatomical and functional changes present in the elderly population, impact upon their health and functionality by generating increased muscle mass, strength, bone density, flexibility, Vo2 max. and static balance, as well as diminished body fat, resting heart rate, and blood pressure, among other aspects^2^. 

The variables determining physical condition are related to movement, that is, a person in good physical shape can perform activities with ease and autonomy; hence, aerobic capacity, body composition, strength, muscular endurance, flexibility, and balance, are essential an individual's health^3^. 

This study sought to identify existing correlations among anthropometric and functional variables and physical exercise habits in a group of elderly adults who regularly attend physical exercise programs. 

##  Materials and Methods

### Participants:

descriptive cross-sectional study, with a population universe of 500 subjects ≥60 years of age, representing 13 regions of Colombia with a mean age of 67.34 (±5.77) years. According to this list of elderly subjects, a representative, probabilistic, proportional sample by sex and delegation was taken, selected at random through a random number table, which comprised 217 participants (58 men and 159 women), with a 5% error margin and 95% confidence index. 

### Instruments:

for the anthropometric evaluation, the following were used: Martin type GMP anthropometer with 200-cm capacity, 1-mm precision, Harpenden fat folds calibrator with an 8-mm capacity, 0.2-mm precision, Mabis anthropometric measuring tape with 150-cm capacity, 1-mm precision, and a Tanita solar scale with 150 kg capacity, with precision from 200 g to 100 kg and 500 g from 100 to 150 kg , using anthropometric techniques globally standardized by the International Society for the Advancement of Kinanthropometry (ISAK)^4^, evaluating body mass, abdominal fat folds, as well as pectoral and anterior thigh (men), triceps, suprailiac, abdominal and anterior thigh (women), waist circumference, and knee height to calculate height. Body density was calculated via the Jackson and Pollock equation^5^ and the percentage of fat via the Siri equation^6^. 

The functional variables were evaluated with ECFA-INEFG tests by Camiña^7^, evaluating abdominal muscle strength with a test of 75 torso flexions during a three-minute period; manual pressure strength through manual dynamometry with a Takei dynamometer Smedly III model; range of articular motion through anterior flexion of the torso with knees extended in sitting position (Wells and Dillon test); postural balance with a monopodal balance test with vision for one minute; Vo2 max from the Rockport test by walking a Fenstermarker mile^8^. In exercise habits, a self-administered questionnaire was used and with support by the study conducted by Osorio *et al*.,^9^. 

### Procedure:

data was collected during the Olympiads for the Elderly, held in Palestina, Caldas, between the 24^th^ and 28^th^ of August 2009. The inclusion criteria were: being older than 60 years of age and having been engaged in a physical exercise program during at least the last year. Authorization was obtained from the bioethics committee from the Faculty of Health Sciences at Universidad de Caldas, complying with regulations in Resolution 8430 of 1993, issued by the Colombian Ministry of Health; permission from delegates and informed signed consent from the participants were also obtained. 

### Data analysis:

the correlation coefficient was determined through Spearman, being the most appropriate method when relating parametric and non-parametric variables, as in this case. Correlation was established with the following criteria: 1.00 functional interrelation, 0.70-0.99 high interrelation, 0.50-0.69 medium interrelation, 0.20-0.49 weak interrelation, 0.09-0.19 very weak interrelation, 0.00 no correlation. Also, a multivariate regression model was made for the Vo2max., as noted in Table 3. Data were analyzed via the SPSS program v. 12. 

## Results

A predominance of women (73%) was found. In the distribution by age groups, there are fewer participants as age increases, as noted in Table 1. 

In the correlation of variables from Table 2, the highest value was presented between BMI and hip circumference. Percentage of fat showed positive correlation with hip and waist circumference. Vo2 max. was negatively associated to percentage of fat and age, but positively with manual pressure strength. 

Torso flexibility was negatively correlated to the waist circumference and age of the individuals. Manual pressure strength was positively correlated to the number of years engaged in sports activities, weekly frequency, and number of hours of physical exercise; likewise, pressure strength was negatively associated to static balance and age. 

Static balance was positively associated to Vo2 max., and negatively associated to age, hip circumference and percentage of fat. This last anthropometric component was negatively associated to the number of hours and weekly sessions of physical exercise. As noted in Table 3, a multivariate regression model was estimated for Vo2 max. All the coefficients of the model (Bi) are significantly different from zero, which indicates that the predictive variables explain Vo2 max; likewise, the global predictive model is highly significant. Additionally, the model fulfills all the assumptions of the classical model (normality of residuals, homocedasticity, and lack of autocorrelation and absence of multicollinearity). 

**Table 1:**
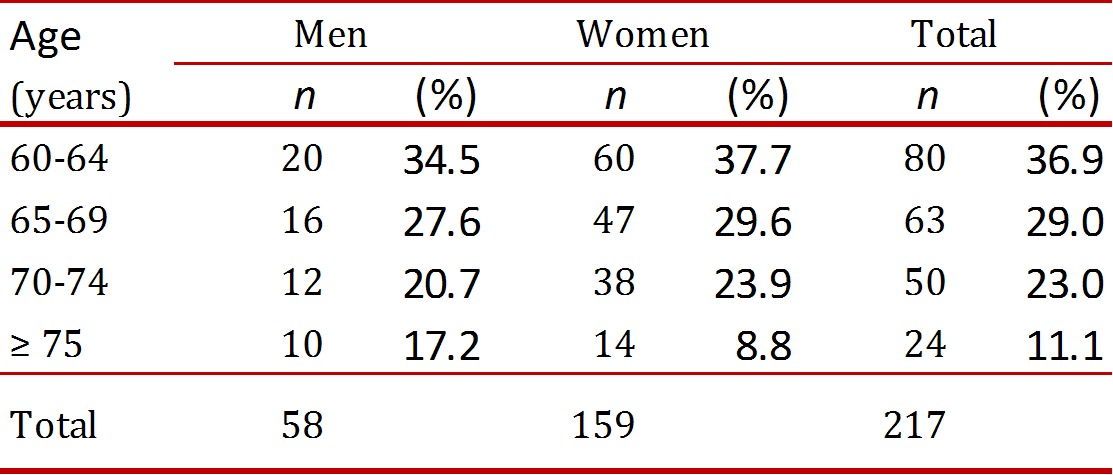
Socio-demographic characteristics of the populatios evaluated

**Table 2.**
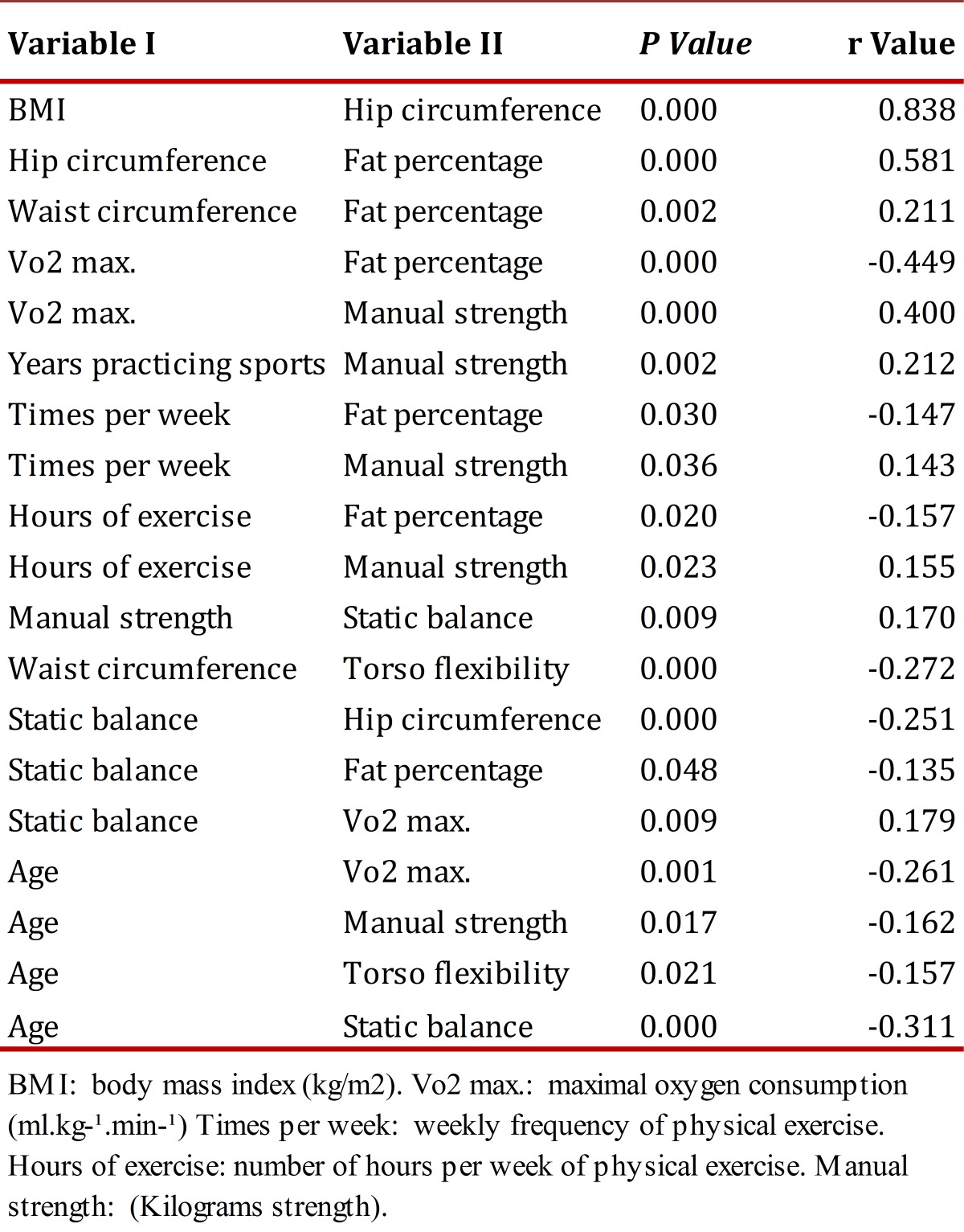
Correlation of Spearman Rho variables, BMI: body mass index (kg/m2).Vo2 max.: maximal oxygen consumption (ml.kg-¹.min-¹)Times per week: weekly frequency de physical exercise. Hours of exercise: number of hours per week of physical exercise. Manual strength: (Kilograms strength)

## Discussion

Percentage of fat was negatively correlated to Vo2 max., with the latter being a predictor of functionality in an individual, which means that an elderly individual with levels of body fat above the normality guidelines, according to Bray's parameters,^10^ from 16 - 19% for men and 21 - 24% for women, will have difficulties in performing basic activities of daily life^11^, which worsens when making efforts that require greater vigor like physical exercise. It must be highlighted that 60.4% of the women and 87.9% of the men in this study had an excess of body fat, which was unexpected, given that the study was conducted with subjects who regularly attended physical exercise programs. 

Adipose tissue is a determining variable in the difference between genders with respect to oxygen consumption, as noted in Table 4, given that a reason why women present less oxygen consumption is precisely because of their higher percentage of body fat^12^. This last aspect was negatively associated to the number of sessions and hours per week dedicated to physical exercise, permitting that inference that with higher weekly frequency and number of hours of physical exercise, the levels of body fat are reduced. 

These previously mentioned findings are coherent with the fundamentals of exercise physiology, where it has been shown that to reduce body fat, calorie intake must be reduced and energy expenditure increased through physical exercise, which - if possible - must be of low intensity, long duration and developed under an appropriate weekly frequency. This is also corroborated by Anton *et al*.,^13^ in a study conducted in the United States with a group of elderly women with overweight problems and functional limitation, who through a six-month structured exercise program significantly reduced fat levels and physical limitations. 

The results of the current study call for reflection upon the pertinence of creating physical exercise programs with greater weekly frequency to that reported by the general average of subjects, given that this factor is related to diminished fat, which contributes to lower risks of functional limitation1 and cerebrovascular disease^14^. 

The BMI was negatively correlated to Vo2 max., hence, subjects with high BMI tend to have lower Vo2 max., given that with greater weight per square meter, there is lower relative oxygen consumption, which is not a particular characteristic among the elderly, as in this instance, but which is also present in young adults, even in high-performance athletes^15^. These causes support the reason why children have higher relative oxygen consumption as compared to adults. 

Hip circumference showed good correlation with BMI, percentage of fat, and waist circumference, variables related to body composition and which permit predicting changes in fat at general levels^16^. These results reveal that hip circumference is determining in predicting functionality in this age group, especially when the results of this variable showed negative correlation with Vo2 max. and static balance. 

Vo2 max. was positively associated to manual pressure strength, given that strength levels are generally related to functional capacity, as with aerobic capacity, including in elderly individuals^17^. This indicates that subjects with good levels of strength have greater ease to carry out activities that imply vigor and energy expenditure, which for this specific case will have enabled their covering a longer distance in the Rockport test; hence, having a favorable Vo2. These results coincide with the study by Osorio *et al*.,^9^ which was also conducted with an elderly population that engages in physical exercise, where a positive correlation was also found between these two variables. 

Regarding the positive correlation among manual pressure strength and the variables of number of years practicing sports activities, amount of sessions and hours per week of physical exercise, diverse authors support the direct relationship among these aspects; initially, Frontera *et al*.,^18^ suggested that for development of muscle strength the frequency of weekly participation is fundamental, given that programs less than three times per week are insufficient to accomplish progress in said aspect. Likewise, Ramírez .^19^ argued that the duration of the processes also plays a relevant role in developing strength, that is, longer time dedicated yields better results. 

Age was negatively associated to manual strength, Vo2 max., flexibility, and balance, considered big predictors of limitation and independence for daily tasks^20^. These results were expected, given that although exercise contributes to slow down organic involution, it is inevitable that with increased age, deterioration occurs in the structural and functional levels, even in individuals who engage in physical exercise. 

Balance influences one of the main dysfunctionality phenomena in elderly adults like falls, which are related to morbidity and mortality in the elderly^21^. It is worth noting that balance was negatively correlated to the percentage of fat and hip circumference, which are related to levels of overweight and obesity in an older population according to Moreno and Ramos^22^, being counterproductive to preserving balance^23^. 

It is important to highlight that the static balance has a positive association with Vo2 max. and manual strength, that is, with lower levels of strength greater probability of losing balance, which is coherent with the findings by Lord with elderly women^24^. This variable was also positively associated to Vo2 max., which indicates that with higher oxygen consumption, there is better capacity to perform activities that imply good functional capacity, with balance as a predictor of that characteristic. 

It can be seen in the regression model for Vo2 max, in Table 3, that with greater age and hip circumference there is lower oxygen consumption, as with greater manual pressure strength this variable increases, which is coherent with findings shown in the correlation of variables, inasmuch as hip circumference and age present negative correlation with Vo2 max., but positive with manual pressure strength. 

These results are supported on theory, which holds that oxygen consumption in human beings diminishes approximately between 5 and 15% per decade as of 30 years of age^25^. Also, the percentage of fat is inversely proportional to oxygen consumption according to Ogawa *et al*.,^12^ which is given in this case through a positive correlation between hip circumference and percentage of fat, coherent with the physiological principles, given that according to the characteristics of the test developed like the Rockport test, an individual with prominent hip circumference will have greater displacement difficulties and, hence, lower oxygen consumption. 

**Table 3:**
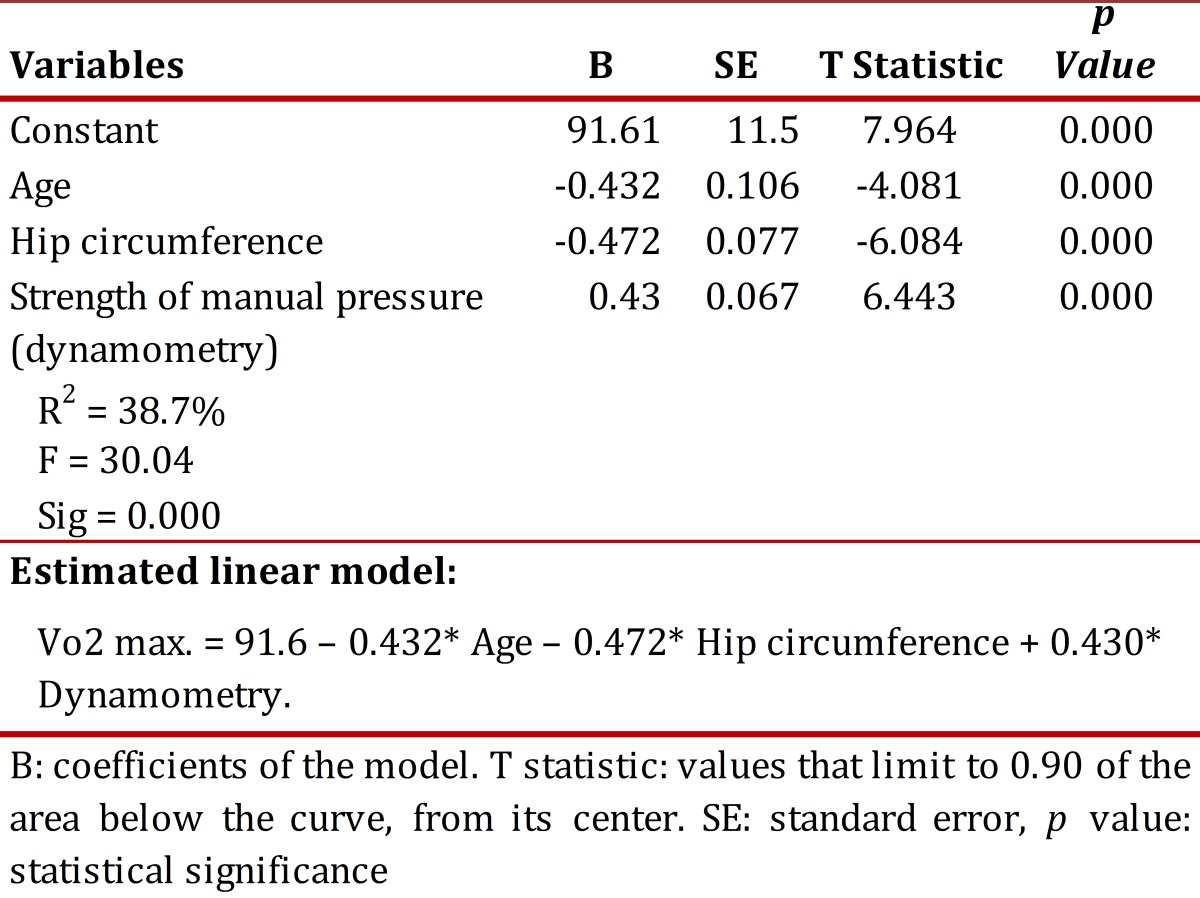
Multivariate regression model for Vo2 max. through the weighted least squares method (LSM), Dependent variable: Vo2 max. SE: standard error, P value: statistical significance. B: coefficients of the model. T statistic: values that limit to 0.90 of the area below the curve, from its center.

**Table 4:**
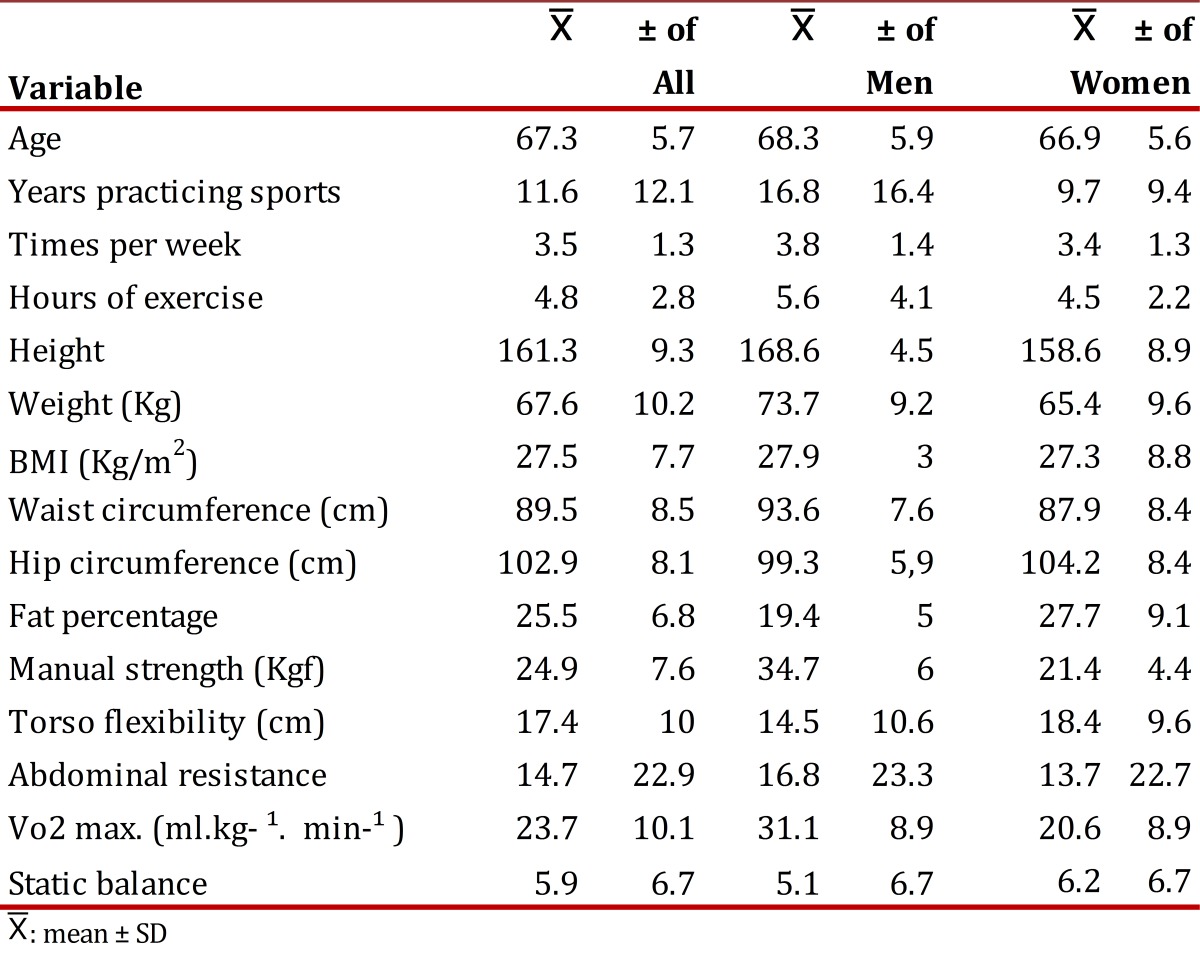
Summary of the mean and standard deviation of the variables evaluated.

## Conclusions

A favorable relationship was observed of physical exercise with increased strength and diminished body fat. 

Although it is true that a negative association was found between age and functional capacity, strength was favorably correlated to engaging in physical exercise, which at the same time was associated negatively to body fat, favoring Vo2 max., balance, and flexibility. 

Strength, aerobic resistance, balance, and flexibility are variables that should be transversally developed and with an appropriate weekly frequency in physical exercise programs to favor the functional capacity of the elderly. 

## References

[1] Arroyo P, Lydia L, Sánchez H,  Bunout  D, Santos JL, Albala C (2007). Indicadores antropométricos, composición corporal y limitaciones funcionales en ancianos.. Rev Méd Chile.

[2] Paz MT (2007). Eficacia de un programa de actividad física municipal en un grupo de adultos mayores físicamente activosDeporte y actividad física para todos. No. Extra5.

[3] González J, Márquez S, Garatachea N, De paz JA, Jimenez R, Bresciani G (2006). Desarrollo de una batería de test para la valoración de la capacidad funcional en las personas mayores (Vacafun-ancianos), y su relación con los estilos de vida, el bienestar subjetivo y la saludUniversidad de León.

[4] Alexander P (2006). International Society for de Advancement of Kinanthropometry (ISAK).

[5] Jackson AS, Pollock ML, Ward A (1980). Generalized equations for predicting body density of women. Med Sci Sports Exerc.

[6] Siri WE, J Brozek JM, Hanschel A (1961). Body composition from fluid space and densityAnalysis of methods. Techniques for measuring body composition.

[7] Camiña F, Cancela CJ, Romo PV (2001). La prescripción del ejercicio físico para personas mayores. Valores normativos de la condición física. Rev. Int. Med.Cienc. aut. Fis. Deporte.

[8] Fenstermarker KL, Plowman SA, Looney MA (1992). Validation of the Rockport fitness walking test in females 65 years and older. Res Q. Exerc. Sport.

[9] Osorio A, Pineda O, Posada L (2002). Características relacionadas con la fuerza de agarre y la resistencia aeróbica en ancianos que realizan ejercicio físico en el estadio de Manizales.

[10] Bray GA, Davison MB, Drenick EJ (1972). A serius symptom. Annals of Internal Medicine.

[11] Honglei Chen, Xuguang Guo (2008). Obesity and functional disability among elder Americans. J Am Geriatr Soc.

[12] Ogawa T, Spina RJ, Martin WH, Kohrt WM, Schechtman KB, Holloszy JO (1992). Effects of aging, sex and physical training on cardiovascular responses to exercise. Circulation.

[13] Anton SD, Manini TM, Milsom VA, Dubyak P, Cesari M, Cheng J (2011). Effects of a weight loss plus exercise program on physical function in overweight,older womena randomized controlled trial. Clin interv Aging.

[14] Correa GI, Benjumea MV (2005). Cómo evaluar el estado nutricional?.

[15] Bergh U, Sjödin B, Forsberg A, Svedenhag J (1991). The relationship between body mass and oxygen uptake during running in humans. Med Sci Sports Exerc.

[16] Hughes V, Roubenoff R, Wood M, Frontera WR, Evans WJ, Fiatarone Singh MA (2004). Anthropometric assessment of 10-y changes in body composition in the elderly. Am J Clin Nutr.

[17] Skelton DA, Young A, Greig CA, Malbut KE (1995). Effects of resistance training on strength, power, and selected functional abilitie of women age 75 and older. J Am Geriatr Soc.

[18] Frontera WR, Meredith CN, O´Reilly KP, Knuttgen HG, Enans WJ (1988). Strength conditioning in older men: skeletal muscle hypertrophy and improved function. J Appl Physiol.

[19] Ramírez JF (2007). El entrenamiento de la fuerza en mayores de 50 años: consideraciones y perspectivas. Arch Med.

[20] Weineck J (2005). Entrenamiento total.

[21] Gómez JF, Curcio CL, Gómez DE (2002). Valoración de la salud de los ancianos.

[22] Moreno BH, Ramos BS (2011). Características antropométricas de adultos mayores participantes en competencias deportivas. Perspect Nutr Hum.

[23] Berrigan F , Simooneau M, Tremblay A, Hue O, Teasdale N (2006). Influence of obesity on accurate and rapid arm movement performed from a standing posture. Int J Obes.

[24] Lord SR, Lloyd DG, Nirui M, Raymond J, Willians P, Stewart RA (1996). The effect of exercise on gait patterns in older womena randomized controlled trial. J Gerontol Biol Sci Med Sc.

[25] Proctor D, Balogobal P, Nair K (1998). Age-related sarcopenia in humans is associated with reduced synthetic rate of specific muscle proteins. J. Nutr.

